# Therapeutic Efficacy and Radiobiological Effects of Boric-Acid-Mediated BNCT in an Osteosarcoma-Bearing SD Rat Model

**DOI:** 10.3390/life13020514

**Published:** 2023-02-13

**Authors:** Chen-Fang Hsu, Hong-Ming Liu, Jinn-Jer Peir, Jiunn-Wang Liao, Kuan-Sheng Chen, Yi-Wei Chen, Yung-Jen Chuang, Fong-In Chou

**Affiliations:** 1 Institute of Nuclear Engineering and Science, National Tsing Hua University, Hsinchu 30013, Taiwan; 2 Nuclear Science and Technology Development Center, National Tsing Hua University, Hsinchu 30013, Taiwan; 3 Graduate Institute of Veterinary Pathobiology, College of Veterinary Medicine, National Chung Hsing University, Taichung 40227, Taiwan; 4 Veterinary Medical Teaching Hospital, College of Veterinary Medicine, National Chung Hsing University, Taichung 40227, Taiwan; 5 Department of Veterinary Medicine, College of Veterinary Medicine, National Chung Hsing University, Taichung 40227, Taiwan; 6 Department of Oncology, Taipei Veterans General Hospital, Taipei 11217, Taiwan; 7 School of Medicine, National Tsing Hua University, Hsinchu 30013, Taiwan; 8 Institute of Bioinformatics and Structural Biology, National Tsing Hua University, Hsinchu 30013, Taiwan

**Keywords:** osteosarcoma, therapeutic efficacy, radiobiological effects, boric-acid-mediated BNCT

## Abstract

Background: Osteosarcoma (OS) is the most common primary malignancy of the bone and is notoriously resistant to radiation therapy. High-dose cytotoxic chemotherapy and surgical resection have improved the survival rate and prognosis of patients with OS. Nonetheless, treatment challenges remain when the tumor cannot be removed by surgery. Boron neutron capture therapy (BNCT) provides high linear energy transfer (LET) radiation, and its internal targeted characteristics make BNCT a novel therapy for removing OS and reducing radiation damage to adjacent healthy tissues. Methods: In this study, a UMR-106-grafted OS rat model was developed, and boric acid (BA) was used as the boron drug for BNCT. The pharmacokinetics of BA, following intravenous injection, were evaluated to determine the optimal time window for neutron irradiation. OS-bearing rats were irradiated by an epithermal neutron beam at Tsing Hua Open-Pool Reactor (THOR). The therapeutic efficacy of and tissue response after BNCT were evaluated by radiographic and histopathological observations. Results: OS-bearing rats were irradiated by neutrons in the first hour following the intravenous injection of BA. The prescription-absorbed doses in the tumor regions were 5.8 and 11.0 Gy. BNCT reduced the body weight of the tumor-bearing rats, but they recovered after a few days. The BA-mediated BNCT effectively controlled the orthotopic OS tumor, reduced osteolysis, and induced bone healing. Autoradiography and histological analysis confirmed that the BA retention region is consistent with the calcification region in OS tissue. Conclusion: BA is specifically retained in OS, and the BA-mediated BNCT can significantly reduce the tumor burden and osteolysis in OS-bearing rats.

## 1. Introduction 

Osteosarcoma (OS) originates from undifferentiated mesenchymal cells that have the ability to differentiate into various types of connective tissue. It is a primary malignant tumor of the bone, that is characterized by the direct formation of an immature bone or osteoid tissue [1]. OS is the most common cancer in adolescents and children due to their rapid skeletal development, and usually starts in the tubular long bones, with 78% occurring in the distal femur and proximal tibia [1].

Although adjuvant chemotherapy with extensive surgery is the standard treatment for OS in the clinic, survival remains around 60–70% after five years and is as low as 30% when pulmonary metastases are detected at diagnosis [2,3]. OS is radioresistant to standard radiotherapeutic doses, therefore radiotherapy is not used for treating this type of tumor. High-dose irradiation may be administered after extensive surgery; however, the postoperative radiotherapy (RT) could damage the normal tissues [4,5]. Owing to osteosarcoma radioresistance, RT treatment for this tumor requires a higher dose than for other malignancies. The administered total RT dose is tailored to the surgical margins and the proximity of organs at risk [6]. The most important issues associated with OS therapy are the improvement of therapeutic efficacy and the reduction of complications. An effective treatment method should have minimal effects on normal tissue and facilitate the rapid recovery of patients. 

Boron neutron capture therapy (BNCT) is an internal targeted radiotherapy, which may be effective in addressing the above issues. This treatment employs sufficient ^10^B that is selectively delivered to the tumor and absorbs thermal neutrons during irradiation. The boron neutron capture nuclear reaction yields high linear energy transfer (LET) α particles (196 keV/μm) and recoiling of ^7^Li (162 keV/μm); these heavy particles have a path length of 5–9 μm in the tissue, and a high relative biological effectiveness [7,8,9]. High LET-charged particle radiation is more cytotoxic per unit dose than low LET radiation, because high LET radiation produces more clustered DNA damage. Clustered DNA double-strand breaks are repaired poorly, so the mutagenic and cytotoxic effects of clustered lesions exceed those of isolated lesions [10,11,12,13].

In BNCT, energy is deposited within the diameter of a single cell, allowing the selective irradiation of the cells. The use of short-range radiation ensures that adjacent normal tissues are spared from radiation-induced damage. Therefore, only neoplastic cells loaded with ^10^B are differentially ravaged following thermal neutron irradiation. The Tsing Hua Open-Pool Reactor (THOR) provides a high-quality neutron beam that can be used for BNCT. More than 330 patients with brain tumors and head and neck cancer have been treated with BNCT at the THOR, with favorable results [14,15,16]. Consequently, the main goal of research on BNCT for OS is to develop an appropriate boron drug. 

In vivo and in vitro studies reveal that boric acid (B(OH)_3_, BA) has a strong affinity for hydroxyapatite in the bone, because it binds to the cis-hydroxy groups [17,18,19]. The ratio of the boron concentration in the blood to that in soft tissue is approximately one, but boron concentrations in bones exceed those in blood by a factor of four, following the oral administration of BA to rats or humans [20,21,22]. Therefore, the purpose of this study is to evaluate the efficacy of BA-mediated BNCT in local tumor control, as well as radiographic and histological responses, using an orthotropic OS model in rats [23].

## 2. Materials and Methods

### 2.1. Preparation of Boric Acid (BA) Solution and Determination of Boron Concentration

^10^B-enriched 99.5% BA was purchased from Stella Chemifa Corporation and used as a boron drug in BNCT to treat osteosarcoma (OS). The BA solution was prepared by adding an adequate amount of BA powder to a normal saline solution, to yield the required ^10^B concentration. The solution was stored as a stock solution (6000 μg/mL) at 4 °C until use. The boron concentration was measured using an inductively coupled plasma atomic emission spectrometer (ICP-AES) [24,25].

### 2.2. UMR-106 Cell Culture

The UMR-106 rat osteogenic sarcoma cell line (BCRC No: 60270) originated from the ^32^P-induced neoplasm in a Sprague-Dawley (SD) rat. Cells were maintained in Dulbecco’s Modified Eagle Medium (Gibco, Waltham, MA, USA), that was supplemented with inactivated 10% fetal bovine serum, 100 U/mL penicillin, and 100 μg/mL streptomycin in a 5% CO_2_ incubator at 37 °C. They were confirmed to be mycoplasma-free by routine testing [23]. 

### 2.3. Animal Model

Four-week-old male SD rats were purchased from BioLASCO Taiwan Co., Ltd. (Taipei, Taiwan). They were bred in an animal room at 22 ± 2 °C and relative humidity of 50–70% with a 12-h light–dark cycle. Unlimited animal feed and water were available, and feeding patterns were monitored. An orthotopic rat model of OS was established mainly following the methods described previously [26,27,28,29]. This model allows for the in vivo imaging and monitoring of tumor growth and was used to develop BA-mediated BNCT strategies. SD rats were anesthetized with 2.5% isoflurane (Halocarbon Laboratories, River Edge, NJ, USA) and the operative field was depilated and disinfected. UMR-106 cells in log-phase growth were harvested and resuspended in a serum-free medium. A 200 μL suspension of cells (1 × 10^7^ cells/200 μL) that was supplemented with a Matrigel matrix (BD Bioscience, 354248, East Rutherford, NJ, USA) was aspirated into a 250 μL fitted, 25-gauge needle, which was inserted by drilling into the femoral condyle of the distal femur. Once the bone was traversed, the needle was inserted further to fracture the posterior cortex of the femur. A 100 μL volume of the cell suspension was injected into the parosteal muscle; another 100 μL was injected into the femoral epiphysis or metaphysis (0.5 cm below the skin surface), while the needle was slowly pulled back. The animals were immunosuppressed by the daily administration of cyclosporin (10 mg/kg, Sandimmun^®^, Novartis, Basel, Switzerland) from day 3 to day 9, following inoculation with the UMR-106 cells. The animal experiments were conducted according to the institutional guidelines that were set (Approval No: 10009) by the Institutional Animal Care and Use Committee (IACUC) of the National Tsing Hua University, Taiwan. All experiments were performed according to sound ethical principles.

### 2.4. Pharmacokinetics and Biodistribution of BA in OS-Bearing SD Rats 

#### 2.4.1. Pharmacokinetic Analysis

Each rat was intravenously bolus-injected with BA (25 mg ^10^B/kg BW) via the tail vein. Blood was sampled at various time intervals to measure the boron concentration by ICP-AES. Pharmacokinetic analysis was conducted to determine the optimal time window for, and duration of, neutron irradiation [24]. 

#### 2.4.2. Biodistribution Analysis 

Rats were intravenously bolus-injected with BA (25 mg ^10^B/kg BW) via the tail vein, and sacrificed at 1, 2 or 3 h after BA injection. Liver, kidney, muscle, intestine, testis, blood, and tumor samples were collected. The samples were weighed and then digested using a microwave digestion system for the subsequent measurement of boron concentration in the tissues, using ICP-AES.

### 2.5. Neutron Irradiation In Vivo

On the 11th day after the tumor cell inoculation, the rats were intravenously injected via their tail veins with 25 mg ^10^B/kg BW of BA, one hour before irradiation. After being anesthetized by a subcutaneous injection with atropine (0.1 mg/kg) and zoletil (0.3 mg/kg), the tumor-bearing legs were irradiated by an BNCT beam at the THOR. The rat holder used for irradiation was made from PMMA (polymethylmethacrylate) and PE (polyethylene) and designed to accommodate three rats irradiated simultaneously. For the rat hind limb irradiation, the osteosarcoma-bearing legs were pulled out of the holder to the beam center. Using the MCNP (Monte Carlo N-particle) simulation code, the physical dose for different parts of the rat were calculated. The rat body and femurs were simulated as a cylinder. The materials in the simulation were defined as specified by the International Commission on Radiation Units Report 46 and the National Institute of Standards and Technology material database.

### 2.6. Histological Analysis

Histological analysis was performed to characterize the response of tissue in the OS-bearing rats to BA-mediated BNCT. UMR-106 cells were inoculated into the femur of SD rats. After 38 days, the rats were sacrificed one hour following BA injection (25 mg^10^B/kg BW). The sampling time, following BA injection, was determined from the pharmacokinetic data. Following sacrifice, the extraskeleton tumor on the legs was collected and processed for paraffin embedding. Embedded specimens were cut into (30 μm thick sections using a microtome (Leica RM 2145, Nussloch, Germany)), and stained with hematoxylin and eosin (Muto Pure Chemicals Ltd., Tokyo, Japan). Alizarin red staining was used to visualize the calcified elements on the tumor slide [30]. An adjacent tumor section was collected for autoradiographic analysis.

### 2.7. Autoradiography 

The localization and microdistribution of boron in tumor-containing tissues of the OS-bearing rat were determined by neutron capture autoradiography. The boron distribution was evaluated by analyzing alpha tracks, using the ImageJ1 software. The neutron autoradiograph was compared to that of a histologically prepared slice. Their fused image was used to determine the distribution of boron in the tissue [31]. 

### 2.8. Radiographic Examination

Radiographic examinations were performed weekly before and after the BNCT. Radiography was carried out on animals that were anaesthetized with zoletil (0.3 mg/kg), using a digital mammographic device (Lorad Selenia, Lorad/Hologic^®^) at the Department of Radiology of the HsinChu Mackay Memorial Hospital, Taiwan. Radiographic images, that are obtained in a fixed mode (24 kVp exposure, 12 mA), wereused to assess the area of an extraskeletal tumor, while those obtained in an auto-filter mode were used to evaluate radiographic grading. The margin of the tumor area was traced to assess the tumor area, using an ImageJ system (ImageJ; National Institute of Mental Health, in. Bethesda, Md.) The tumor area in a rat in each group was compared with that of the same rat on the day of the BNCT treatment to yield the relative tumor size. 

### 2.9. Statistical Analysis

Data are expressed as the mean ± SD. The student’s two-tail t-test was used to compare the parameters of the groups. The difference between the control and treated groups was regarded as statistically significant at *p* < 0.05.

## 3. Results

### 3.1. Biodistribution of Boron in Osteosarcoma-Bearing Rat

**Figure 1:**
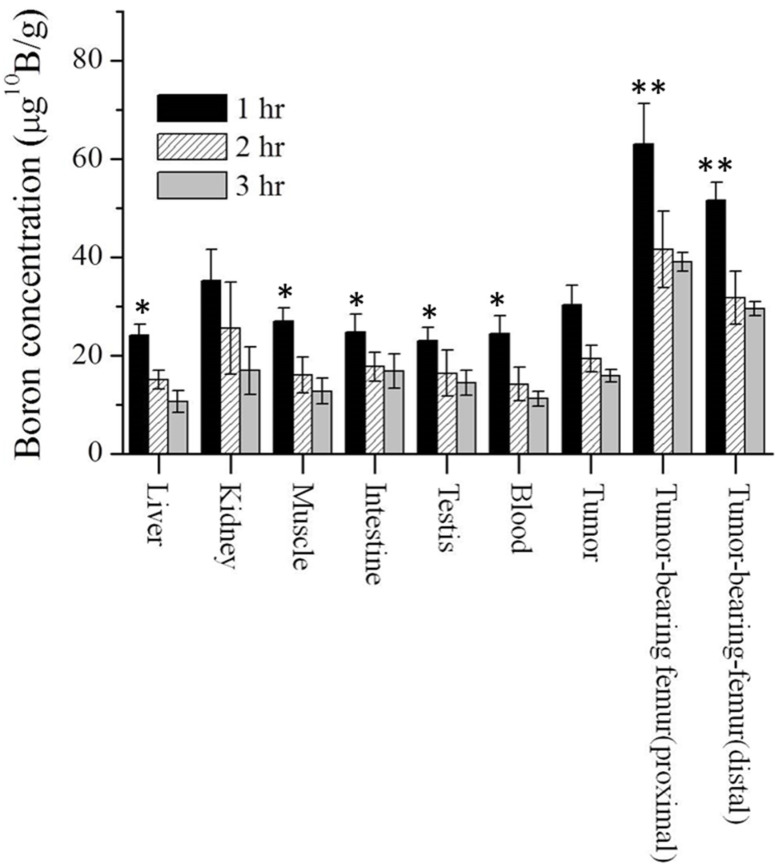
The boron concentrations in major organs, extraskeletal tumor tissue and tumor-bearing bone in the OS-bearing rats. The rats received a tail vein injection of boric acid at a dose of 25 mg ^10^B/kg body weight. The organs and tissues were sampled at 1, 2 and 3 h after the boric acid injection. The tumor to muscle ratio was 1.34. Each bar represents the mean ± SD (*n* = 3–5, ** *p* < 0.01 relative to tumor, * *p* < 0.05 relative to tumor).

The boron concentration in the major organs, and various tumor tissues was assayed one, two, and three hours after the injection of boric acid on the 11th day, following tumor implantation. Figure 1 shows these boron concentrations. The boron concentrations in soft tissue were similar to that in the blood. At one hour after administration, the boron concentrations in the blood, extraskeletal tumor, tumor-bearing proximal femur, and the tumor-bearing distal femur were 22.1 ± 3.5, 32.5 ± 3.9, 63.2 ± 8.2, 51.6 ± 3.7 μg ^10^B/g, respectively; at two hours after administration, the corresponding values were 14.1 ± 3.4, 19.3 ± 2.7, 41.5 ± 7.8, 31.7 ± 5.4 μg ^10^B/g, respectively; and at three hours after administration, the values were 11.2 ± 1.5, 15.8 ± 1.3, 39.0 ± 1.9, 29.5 ± 1.4, respectively. The ratios of the boron concentrations in the extraskeletal OS tumor, tumor-bearing proximal femur, and tumor-bearing distal femur to that in blood were 1.5 ± 0.4, 2.8 ± 0.5, and 2.3 ± 0.4 at one hour after administration, respectively.

### 3.2. Physical Dose for BNCT

UMR-106 cells were injected into the femur of SD rats. On day 11 following inoculation, the tumor-bearing rats were separated into three groups: the tumor control group, the high-dose, and the low-dose BNCT groups (*n* = 3–5). These rats were intravenously injected via their tail veins with 25 mg ^10^B/kg BW of BA, one hour before irradiation. After the rats were anesthetized, they were fixed in a holder and irradiated with an epithermal neutron beam. The actual reactor power and irradiation time were corrected using an online neutron monitoring system. Table 1 shows the physical doses that correspond to 0.5 and 1 h of neutron irradiation in the low-dose and high-dose BNCT groups, respectively. In the high-dose BNCT group, the tumor-bearing femurs received a total dose of 15.1 Gy, which includes the neutron dose (1.11 Gy), the gamma dose (1.95 Gy), and the boron dose (12.03 Gy), corresponding to 7.4%, 12.9% and 79.7% of the total physical dose, respectively. The rat body and tumor received 2.43 and 11.03 Gy during irradiation, respectively.

The physical doses that correspond to 0.5 h (low-dose BNCT group), and 1 h (high-dose BNCT group) of neutron irradiation are shown in Table 1.

### 3.3. Effect of BNCT on Body Weight

**Figure 2:**
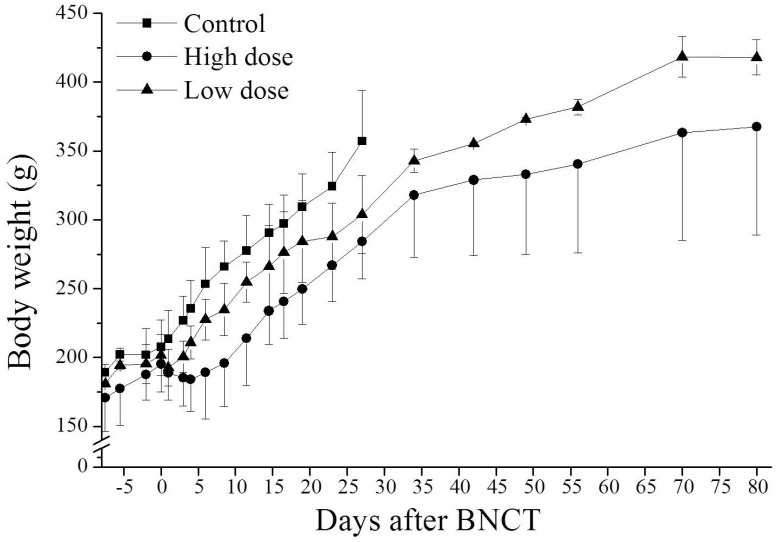
Body weight of OS-bearing rats within 80 days following BNCT. In the high-dose BNCT group, the tumor regions of OS-bearing rats received a dose of 11.0 Gy; and in the low-dose BNCT group, the tumor regions of OS-bearing rats received a dose of 5.8 Gy. Each point represents the mean ± SD (*n* = 3–5).

Figure 2 plots the dose-related changes in the body weights of rats as a result of two doses of BNCT. Animals in the high-dose BNCT group lost weight and those in the low-dose BNCT group stopped gaining weight as a result of BNCT. In the low-dose group, each rat started gaining weight on the 3rd day following BNCT; in the high-dose BNCT group, each rat started gaining weight on the 8th day after BNCT. The body weights of BNCT-treated rats changed due to diarrhea and loss of appetite. The body weight of the tumor-bearing control rats increased up to day 35, after the implantation of tumor cells, and decreased thereafter. The results reveal that the weight loss as a result of irradiation was a temporary side effect of BNCT, and the lost weight was recovered thereafter.

### 3.4. Therapeutic Efficacy

#### 3.4.1. BNCT Reduces Size of Tumor

**Figure 3:**
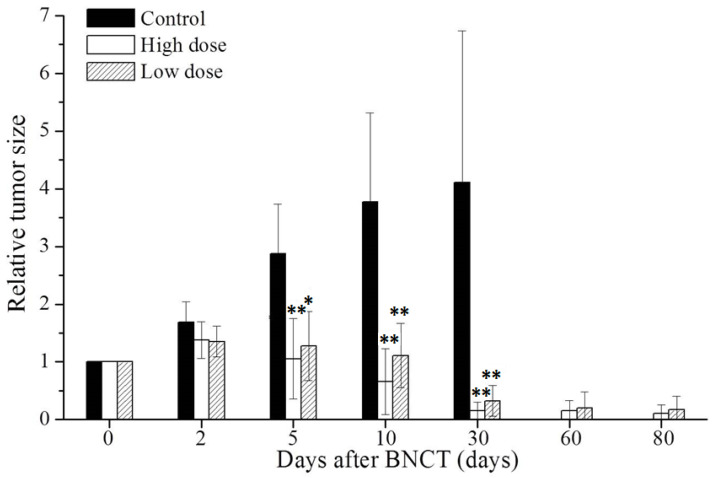
Relative extraskeletal tumor areas before and after BNCT treatment in OS-bearing rats. In the high-dose BNCT group, the extraskeletal tumor regions received a dose of 11.0 Gy; and in the low-dose BNCT group, the tumor regions received a dose of 5.8 Gy. The tumor region was traced using radiography within 80 days after BNCT. The relative tumor size was calculated as the tumor area after BNCT, divided by the tumor area on the day of BNCT. Each bar represents the mean ± SD (*n* = 3–5, ** *p* < 0.01 relative to controls, * *p* < 0.05 relative to controls).

The therapeutic efficacy of BNCT was evaluated from the changes in the tumor areas that were determined by radiographic examination. Relative tumor size was calculated as tumor area after BNCT, divided by tumor area on the day of BNCT. Figure 3 displays the size reduction of extraskeletal tumor, as a result of BNCT. The relative tumor sizes on day 5 were 2.9, 1.1, and 1.3 in the tumor control, high-dose, and low-dose BNCT groups, respectively; on day 10, the relative tumor sizes were 3.8, 0.7, and 1.1, respectively; and on day 30, the relative tumor sizes were 4.1, 0.2, and 0.3, respectively. In the tumor control group, the OS-bearing rats were sacrificed for large tumors, and the tumor sizes were measured on day 30. The relative tumor areas in the high-dose and low-dose BNCT groups on day 80 were 0.1 and 0.2, respectively. The relative tumor areas in the BNCT group and the control group on day 5 differed significantly (*p* < 0.05). The rate of reduction of the tumor size gradually declined within 30 days following BNCT treatment, and the tumor sizes were reduced continually without recurrence in 80 days.

#### 3.4.2. Radiographic Investigation

**Figure 4:**
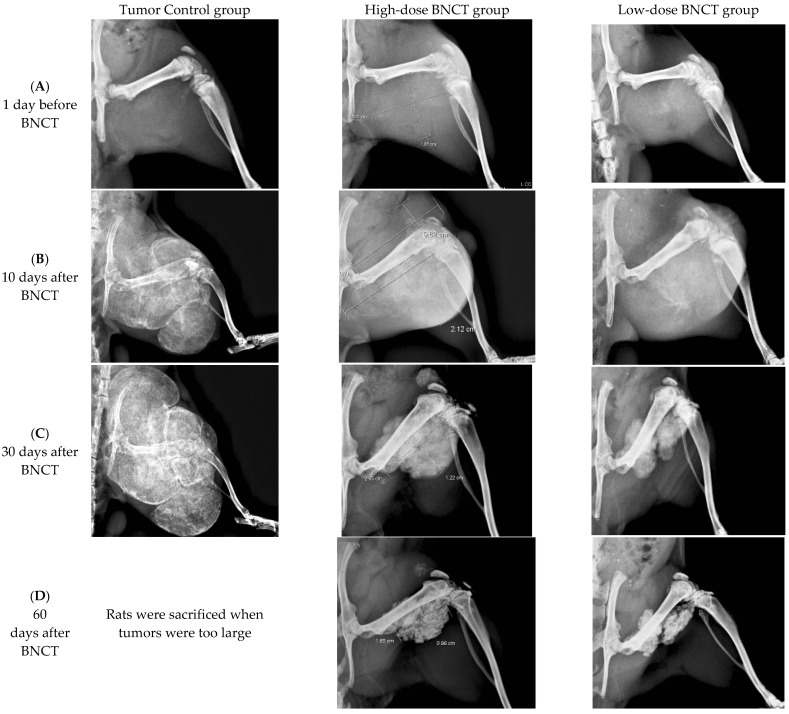

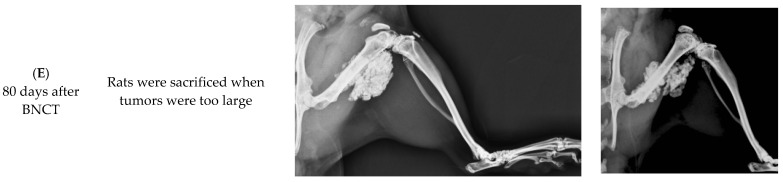
Radiographic investigation of BA-mediated BNCT rats. Radiographs taken of rats in the high-dose BNCT group and the low-dose BNCT group were obtained on the day before BNCT and the 10th, 30th, 60th, and 80th days after BNCT, respectively. The OS-bearing rats in the tumor control group were sacrificed and data were collected until day 30.

Radiographs of the hind limbs of rats were obtained. Figure 4 displays the radiographs of the tumor control, high-dose-treated and low-dose-treated rats on the day before BNCT and 10, 30, 60, and 80 days following BNCT treatment. The reductions of the tumor sizes in the BNCT-treated groups were investigated, and increases in tumor sizes in the tumor control group were observed. The tumor sizes declined significantly on the 30th day following BNCT. Inhibition of tumor growth and tumor shrinkage were apparent when tumor cells were injured as a result of BNCT. By day 80 after BNCT, high-radiodensity scar tissue had been formed by the shrinkage of the large tumors in both high-dose and low-dose BNCT groups. Evidently, BA-mediated BNCT significantly and rapidly shrank the extraskeletal tumor.

On the 10th day following BNCT, a small radiolucent lesion was present on the posterior site of the injection in the tumor control group. On day 30 after BNCT, the radiolucent lesion was shown on the posterior site of the injection, and the radiolucent lesion on the anterior site of injection was also increased. The progressive radiolucent lesion was extending in the tumor control group and the rat was sacrificed when the tumor was too large.

### 3.5. Relationship between BA Retention and Calcification in OS Tissue

**Figure 5:**
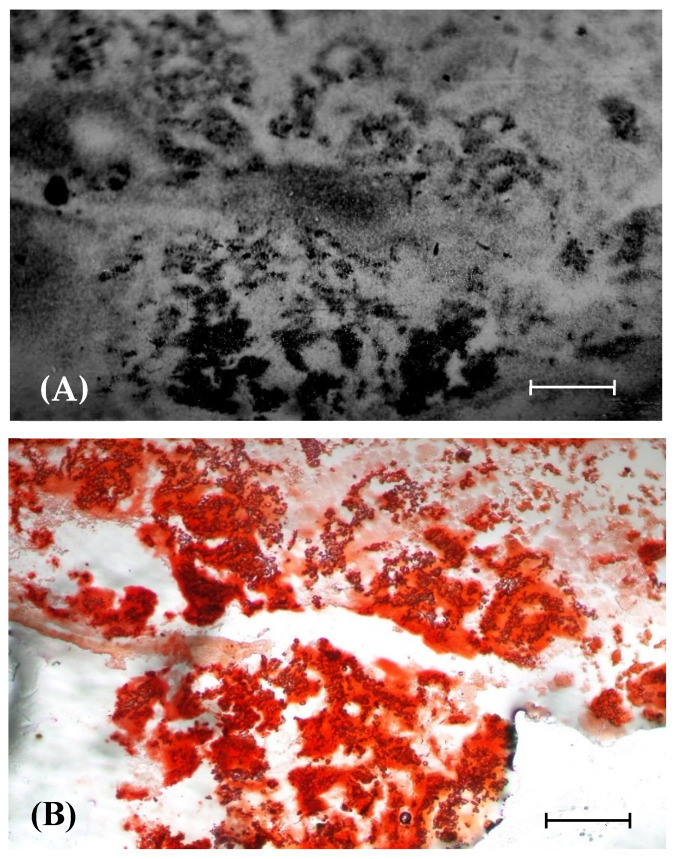
Autoradiography and histological observation of OS tissue section. (**A**) Alpha-track autoradiographic image of an extraskeletal tumor section; (**B**) corresponding Alizarin-red-stained histological section. Tissue sample: UMR-106 cells were inoculated into the femur of an SD rat. After 38 days, the rat was sacrificed one hour after the BA injection (25 mg ^10^B/kg BW). Scale: 1 mm.

The relationship between BA retention and calcification in OS tissue was investigated using autoradiography and Alizarin red staining. The spatial boron distribution and calcified deposits in OS tissue were analyzed. Figure 5 presents autoradiographic images of a slice of OS tissue and the corresponding Alizarin-red-stained histological section. The high-density dark zones correspond to high ^10^B concentrations in the alpha-track autoradiographs, and these were compared to the Alizarin-red-stained regions of the histological section. The microdistribution of the alpha tracks in the tumor regions was heterogeneous. The region of high-density alpha tracks corresponds closely to that of Alizarin-red-positive deposits. The BA retention area in OS tissue is consistent with the calcified area.

## 4. Discussion

In this study, boric acid (BA) was used as a boron-containing drug, and an animal model of OS-bearing rats was used to prove that BNCT was effective for treating OS. Owing to the high radiation resistance of OS, the potential toxic effect of conventional radiotherapy on adjacent structures or organs is an issue. BNCT is an internally targeted radiotherapy, and the distribution of radiation that is delivers is determined by the selective accumulation and microdistribution of the boron drug in the tumor. BNCT can hence overcome the shortcomings of traditional radiotherapy and is effective for treating OS. 

BA-mediated BNCT is not only an effective method for treating OS, but also has advantages in the treatment process. In BA-mediated BNCT, the boron-containing drug (BA) is administered intravenously to the patient. The patient’s affected part (tumor) does not need to be surgically removed for ex vivo irradiation [32], as the tumor is irradiated with neutrons in situ. 

Under conventional radiotherapy, the delivery of high doses to target volumes in OS may be greatly limited by the usually large size of the primary tumors and their frequent localization in anatomically challenging regions, such as the pelvis or the paraspinal area [5,6]. However, the affected region can be treated multiple times in BNCT. Since BNCT is an internally targeted radiotherapy, it provides a favorable dose distribution, allowing the administration of radiation to tumors near or in anatomically challenging regions. The high LET radiation, that is generated by the boron neutron capture reaction at tumor sites, may overcome the radiation resistance of OS cells. Moreover, OS is most often found in long bones, mostly in the legs and sometimes in the arms. In BNCT, the highest possible dose could be administered to the tumor, given the nearby organs could be protected. When an OS undergoes BNCT, since no critical organ is close to the long bone, the affected region can be given a high dose of irradiation, effectively killing the tumor cells. 

In clinical BNCT in the THOR, the blood of the patient is sampled and the boron concentration is evaluated at one and two hours during the boron-drug infusion. The boron concentration in the blood sample in the second hour was one of the operating parameters that is input to the on-line monitoring system for dose calculation. Then, the power is set and neutron beam irradiation is initiated. Since BA does not specifically accumulate in the soft tissues of a living creature, and the concentration of boron in a patient’s blood is approximately that of the patient’s soft tissues, thus, the blood boron concentration can be used to calculate the limiting dose in normal tissues around the tumor, and to estimate the dose that is received by the tumor.

Our prior studies have shown that the distribution of Alizarin-red-stained positive regions in histological sections were similar to that of high-density alpha tracks in the autoradiographs. A perfect co-localization between the regions of high ^10^B concentration and Alizarin-red-positive deposits was observed (Figure 5), revealing that the deposition of BA in OS tissue is related to its calcification activity. In vivo and in vitro studies indicate that boric acid (B(OH)_3_, BA) has a strong affinity with hydroxyapetite in the bone, owing to its binding to the cis-hydroxy groups [17,18,19]. OS cells are characterized by the formation of calcified tissues from osteoids. More BA, specifically, accumulates in the osteoids of tumor tissues than in the soft tissues of a living creature. The effect of boric acid on cartilage formation of osteochondral defects in rabbit knee has been reported [33]. Additionally, the vasculature of a tumor is different from that of normal tissue, and it may take up more BA than normal vasculature. Therefore, when an OS undergoes neutron irradiation, the vasculature may suffer more damage than that of normal tissue [24,34]. 

In this OS-bearing animal model, lung is the most frequent metastatic site. On the 15th day after tumor cell inoculation, some rats died due to lung metastasis. Lung histological examination showed that the tumor was nodular, with round-to-spindle-shaped tumor cells, high degree of mitosis, and a few osteoid giant cells. Once osteosarcoma cells invaded the lungs, SD rats quickly lost weight and had difficulty breathing, and even died. On the 41st day after tumor inoculation, OS-bearing rats partly died due to lung metastasis and the rest were humanely sacrificed, because the diameter of the in situ tumors exceeded 4 cm. There were no surviving rats in the untreated group.

In this study, the UMR-106 cell suspension was injected into the parosteal muscles and distal femoral epiphysis or metaphysis. Thus, OS formed in the distal femur and extraskeletal tumors in the parosteal muscles. These tumor models were used to study the therapeutic and radiobiological effects of boric-acid-mediated BNCT. BA-mediated BNCT was verified to have the potential of treating OS of the femur, by shrinking the tumor and healing the bone. BA selectively accumulates in OS tissue, and is involved in tissue calcification during the development of OS. The rapidly grown Os produced more osteoid in tissues, causing the accumulation of more BA in the tumor tissue. However, as shown in Figure 4, the low-dose BNCT group does not appear to be significantly different from the high-dose BNCT group, possibly indicating that low-dose BNCT has sufficient therapeutic effect on OS. The clinical relevance of each entity requires further study.

## 5. Conclusions

The results of this study indicate that BA can be specifically retained in OS, revealing that the deposition of BA in OS tissue is related to its calcification activity. The radiobiological effects of BA-mediated BNCT have a temporary side effect and can be recovered, thereafter. Thus, BA-mediated BNCT significantly reduces the tumor burden and osteolysis in the OS-bearing SD rats.

## Figures and Tables

**Table 1:** The physical dose components of BNCT delivered to tumor and tumor-adjacent tissue in osteosarcoma-bearing rat.

Organ	Low-Dose BNCT Group (Gy) (% of Total Dose)				High-Dose BNCT Group (Gy) (% of Total Dose)			
	Total Dose	Neutron Dose	Gamma Dose	Boron Dose	Total Dose	Neutron Dose	Gamma Dose	Boron Dose
Rat body	1.27	0.14 (11%)	0.37 (29 %)	0.76 (60 %)	2.43	0.26 (11%)	0.71 (29 %)	1.46 (60%)
Tumor-bearing femur	7.90	0.58 (7%)	1.02 (13%)	6.30 (80%)	15.09	1.11 (7%)	1.95 (13%)	12.03 (80%)
Extraskeletal Tumor	5.77	0.82 (14%)	1.20 (21%)	3.76 (65%)	11.03	1.56 (14%)	2.30 (21%)	7.18 (65 %)
Testes	3.30	0.44 (13%)	0.89 (27%)	1.97 (60%)	6.30	0.85 (13%)	1.70 (27%)	3.76 (60%)
Intestine	3.01	0.24 (8%)	0.84 (28%)	1.93 (64%)	5.99	0.49 (8%)	1.67 (28%)	3.83 (64%)

## Data Availability

All the data supporting the conclusions of this article are included within the article.
